# Hemocompatibility improvement of perfusion-decellularized clinical-scale liver scaffold through heparin immobilization

**DOI:** 10.1038/srep10756

**Published:** 2015-06-01

**Authors:** Ji Bao, Qiong Wu, Jiu Sun, Yongjie Zhou, Yujia Wang, Xin Jiang, Li Li, Yujun Shi, Hong Bu

**Affiliations:** 1 Laboratory of Pathology, West China Hospital, Sichuan University, Chengdu, 610041, China; 2 Department of Pathology, West China Hospital, Sichuan University, Chengdu, 610041, China; 3 Key Laboratory of Transplant Engineering and Immunology, Ministry of Health, West China Hospital, Sichuan University, Chengdu, 610041, China; 4 Department of General Surgery, The first people’s hospital of Yibin, Yibin, 644000, China; 5 College of Polymer Science and Engineering, Sichuan University, Chengdu, 610041, China

## Abstract

Whole-liver perfusion-decellularization is an attractive scaffold–preparation technique for producing clinical transplantable liver tissue. However, the scaffold’s poor hemocompatibility poses a major obstacle. This study was intended to improve the hemocompatibility of perfusion-decellularized porcine liver scaffold via immobilization of heparin. Heparin was immobilized on decellularized liver scaffolds (DLSs) by electrostatic binding using a layer-by-layer self-assembly technique (/h-LBL scaffold), covalent binding via multi-point attachment (/h-MPA scaffold), or end-point attachment (/h-EPA scaffold). The effect of heparinization on anticoagulant ability and cytocompatibility were investigated. The result of heparin content and release tests revealed EPA technique performed higher efficiency of heparin immobilization than other two methods. Then, systematic *in vitro* investigation of prothrombin time (PT), thrombin time (TT), activated partial thromboplastin time (APTT), platelet adhesion and human platelet factor 4 (PF4, indicates platelet activation) confirmed the heparinized scaffolds, especially the /h-EPA counterparts, exhibited ultralow blood component activations and excellent hemocompatibility. Furthermore, heparin treatments prevented thrombosis successfully in DLSs with blood perfusion after implanted *in vivo*. Meanwhile, after heparin processes, both primary hepatocyte and endothelial cell viability were also well-maintained, which indicated that heparin treatments with improved biocompatibility might extend to various hemoperfusable whole-organ scaffolds’ preparation.

Liver transplantation is currently the only effective treatment for end-stage liver diseases. However, the shortage of donor organs limits the success of this approach. Liver engineering is a potential alternative to allotransplantation for end-stage liver failure. Decellularization is an attractive technique for scaffold preparation as the resulting material can potentially retain the architecture of the original tissue^1^. The potential applications of decellularized matrix in tissue engineering have been demonstrated for a number of tissues, including bladder^2^, urethra^3^, skin^4^, trachea^5^, artery^6^, and esophagus^7^. Whole-organ scaffolds can be created by detergent perfusion via the native vasculature, generating an acellular matrix suitable for recellularization with selected cell types. Perfusion decellularization of cadaveric livers removes cells and generates a cell-free extracellular matrix scaffold containing acellular vascular conduits, which are theoretically sufficient to perfuse and support tissue-engineered liver constructs. We have constructed a portal implantable functional tissue-engineered liver using a decellularized liver scaffold (DLS) and hepatocytes in rats^8^. Recently, this process has been scaled up, generating biocompatible scaffolds at a clinically relevant scale^9^. Although heterotopic transplantation of these acellular scaffolds is possible, the scaffolds remain thrombogenic, even with anti-coagulation following transplantation^10^. The thrombogenicity of the scaffold is one of the main obstacles to research on engineering clinical transplantable liver tissue^11^.

Surface-immobilized heparin on collagenous tissue has been reported to prevent thrombus formation^12^. We have developed a layer-by-layer (LBL) self-assembly heparin-coating technique to electrostatically immobilize heparin on rat DLSs^8^. Wang *et al.*^13^ reported that heparin can be covalently immobilized on collagenous tissue by activating its carboxyl groups using a water-soluble carbodiimide via multi-point attachment (MPA). In addition, Larm *et al.*^14^ developed a procedure in which nitrite-depolymerized heparin may be covalently immobilized on polyurethane tubing via end-point attachment (EPA). Heparin covalently immobilized on the surface of biomaterials through MPA or EPA has been shown to retain its antithrombogenic activity^13^,^14^.

The present study was intended to provide a method for producing blood compatibility with DLSs. We immobilized heparin using LBL electrostatic immobilization (Fig. 1A-left) and covalent immobilization via MPA (Fig. 1A-middle) or EPA (Fig. 1A-right). We evaluated the performance of the heparinized scaffolds by measuring immobilized heparin contents and release rates, coagulation time, platelet adhesion and activation, cell culture *in vitro* and implantation *in vivo* (Fig. 1B). These hemocompatibility improvement studies are the first step toward generating an engineered transplantable functional liver decellularized whole organ scaffolds.

Hereafter, /h-LBL, /h-MPA, and /h-EPA refer to DLSs with heparin immobilized using the LBL, MPA and EPA techniques, respectively. Non-heparinized DLS was used as control.

## Results

### Perfusion decellularization of porcine livers

Twenty-two whole livers were perfusion decellularized successfully out of 24 isolated livers from mini-pigs. The perfusion whole-organ decellularization process yielded a fully decellularized porcine liver with a translucent appearance (see Supplementary Fig. S1A online). Histological evaluation revealed no remaining nuclei or cellular material throughout the entire thickness of the graft (see Supplementary Fig. S1B online). 4,6-diamidino-2-phenylindole (DAPI) staining (see Supplementary Fig. S1C online) and scanning electron microscope (SEM) analysis (see Supplementary Fig. S1D online) showed that the liver cells were completely removed, whereas the collagen and fibers were maintained. The remnant DNA extract was subjected to agarose gel electrophoresis to reveal the length of DNA fragments in the decellularized liver scaffold (DLS), which can barely be seen by visual inspection (see Supplementary Fig. S2A online). The DNA content in the normal tissue was 9.1 ± 0.2 μg/mg, whereas the DNA content in DLS was 0.03 ± 0.01 μg/mg (see Supplementary Fig. S2B online).

### Properties of DLSs

Histological and immunohistochemical staining revealed that, compared to normal liver tissue, sulfated glycosaminoglycans (GAGs), collagen fibers, elastic fibers, and reticular fibers were well retained (see Supplementary Fig. S3A online) and ECM components, including collagen I and IV, laminin, and fibronectin, were preserved in DLSs (see Supplementary Fig. S3B online). The collagen content was almost 8-fold higher DLSs than in normal liver tissue (see Supplementary Fig. S3C online). Moreover, nearly 70% of the GAGs were retained in DLSs compared to normal liver tissue (see Supplementary Fig. S3D online).

### Elimination of xenogeneous antigens in DLSs

The porcine DNA sequences of porcine endogenous retrovirus (PERV), PERV polymerase (PERV Poly), swine leukocyte antigen DR alpha (SLA-DRA), swine leukocyte antigen 2 (SLA-2), sus scrofa cytochrome b (SsCytb) and, porcine beta actin (β-actin) were detected in native organs. Otherwise, these DNA sequences were absent in the ECM after decellularization (Fig. 2A). The immunohistochemistry for α-Gal showed although native materials were found to be Gal positive, the most remarkable reduction of the Gal epitope can be observed in the DLSs (Fig. 2B).

### Homogeneous heparin immobilization on the DLSs

To compare the efficiency of three heparinized methods, the different heparin solutions with the same heparin concentrations (1, 0.5 or 0.25 g/L) were perfusion through portal vein (PV) at the same flow rate (100 mL/min) for 4 h in total. Analyzed samples were taken from 12 different sites (S1 to S12) as shown in Fig. 3A, which involved the porta hepatis to margin of the right, median and left lobes of the whole liver. Based on the heparin content results of S1 to S12 from each liver with different heparin treatments, the coefficients of variations (CV%) for three methods were 4.09% (LBL), 1.09% (MPA) and 1.63% (EPA) respectively at 1 g/L heparin preparation (Fig. 3B). Table 1 shows three types of heparinized scaffolds gained different contents of heparin immobilization. The /h-EPA scaffold gained the highest heparin content (*p* < 0.001) at 1 g/L heparin preparation. Heparin–toluidine blue (TB, C.I. Basic Blue 17) interaction was employed as analysis of functional groups present on the polyanion chain. The colorimetric assay of heparin by means of metachromatic dyes TB is easy and quick. The heparinized DLSs showed even and positive staining with TB throughout the entire matrix, whereas control DLSs did not stain with TB (Fig. 3C). Compared with the /h-LBL and /h-MPA scaffolds, the inside of /h-EPA scaffolds were more deeply stained. SEM imaging showed that the surface morphology of /h-EPA group DLSs was similar to the non-heparinized DLSs (Fig. 3D).

However, to compare the surface properties of the tissues immobilized with heparin using different methods impartially, the amounts of heparin immobilized on all test samples were intentionally adjusted to be approximately the same via shorten the heparin treatment time of /h-LBL and /h-EPA scaffolds. Hereafter, the heparin contents of /h-LBL, /h-MPA, and /h-EPA scaffolds were 10.36 ± 0.06, 10.25 ± 0.08 and 10.51 ± 0.05 μg/mg dry weight, respectively, for the following experiment tests.

### /h-EPA scaffold gained the lowest heparin release rate

Heparin accumulative release (Fig. 4A) and sustained release (Fig. 4B) tests showed that heparin was quickly released from the /h-LBL, /h-MPA, and /h-EPA scaffolds on day1, followed by slow release for 7 days. After 7-day accumulativer or sustained release test, above 33% or 42% of heparin retained in /h-EPA scaffolds, compared with original heparin content, respectively.

### Anticoagulation abilities improved significantly in heparinized DLSs

PT, TT and APTT have been applied for evaluation of the *in vitro* anti-thrombogenicity of biomaterials^15^. The ranges of PT for control, /h-LBL,/h-MPA and /h-EPA scaffolds were 10.9 ± 0.7, 38.4 ± 1.1, 35.3 ± 0.9 s and 137.5 ± 15.6 s, respectively (Fig. 5A). The values of TT for control, /h-LBL, /h-MPA and /h-EPA scaffolds were 17.0 ± 1.1, 24.8 ± 1.2, 23.3 ± 0.9 s and 125.6 ± 10.6 s, respectively (Fig. 5B). The ranges of APTT for control, /h-LBL, /h-MPA and /h-EPA scaffolds were 23.1 ± 1.2, 80.3 ± 2.8, 60.6 ± 3.9 s and 375.2 ± 28.2 s, respectively (Fig. 5C). The TT, PT, and APTT of heparinized scaffolds were obviously longer than those of the control group.

### Heparinized treatments reduced platelet adhesion and activation

Platelet adhesion studies were conducted with the purpose of determining the blood compatibility of each test sample, because platelet adhesion is one of the most important procedures during blood coagulation on prosthetic surfaces^16^. Field counts of adherent platelets were made using representative micrographs at similar magnifications to meter the differences observed using SEM. Fig. 6A shows examples of SEM micrographs of platelets adhered to test samples. The number of platelets adhered to all test scaffolds were counted and are compared in Fig. 6B. The results showed that the heparinized DLSs retained significantly fewer platelets than the control scaffolds (*p* < 0.05). In addition, fewer platelets adhered to the /h-EPA scaffolds (117 ± 13 platelets/15000 μm^2^) than to the /h-LBL (177 ± 14 platelets/15000 μm^2^) and /h-MPA scaffolds (163 ± 11 platelets/15000 μm^2^) (*p* < 0.05). However, no significant differences were observed between the /h-LBL and /h-MPA scaffolds (*p* > 0.05).

Platelet factor 4 (PF4) was used to detect platelet activation by modified biomedical materials^17^. Figure 6C shows the PF4 concentrations in plasma after the scaffolds contacted with blood; it could be observed that for the heparinized DLSs the PF4 concentration showed a prominent decrease compared with normal plasma or control DLS. Combined with the numbers of the adhered platelets, these results indicated that platelet activation on the heparin treated DLSs was greatly suppressed.

### *In vivo* implantation of DLSs

The mechanical properties of the vasculature deprived of the endothelial layer supported the surgical reconnection of the vessel stumps of the scaffold to the recipient’s renal vein (RV) and splenic vein (SV). After connection with RV, blood flowed well within the whole scaffold (Fig. 7A). DLSs were filled with blood within 5 min, which swelled gently and acquired a color similar to normal livers (Fig. 7B). Satisfactory blood flow throughout the heparinized DLSs was observed for 60 minutes (Fig. 7C), whereas no blood flow throughout the control DLSs was observed after 20 min perfusion. No leaks were detected and no adverse events were recorded during surgery. At the end of perfusion, the graft was harvested for histologic analysis. H&E staining revealed vascular structures of non-heparinized DLSs were completely occluded by thrombosis after 20 min blood perfusion, whereas vascular trees of other heparinized DLSs were still open after 60 min perfusion (Fig. 7D).

### Heparinized DLSs maintained good cellular compatibility

Rat primary hepatocytes and human umbilical vein endothelial cells (HUVECs) were cultured *in vitro* to measure the cytocompatibility of the heparinized DLSs. H&E staining revealed that primary hepatocytes aggregated together to form multi-layer cell structure after 3 days seeded on both control and heparinized DLSs (Fig. 8A). Immunohistochemical analysis of cell proliferation showed that approximately 80% of the cultured cells stained positive with proliferating cell nuclear antigen (PCNA) antibody (Fig. 8B). Morphologies of the HUVECs cultured for 3 days on the control and heparinized DLSs are shown in Fig. 8C. It was observed that the HUVECs exhibited a typical flattened morphology on the scaffolds. Immunohistochemical analysis of cell proliferation showed that approximately all of the cells stained positive with PCNA (Fig. 8D). The results indicated heparinized DLSs could support cell adhesion and growth as the same as control scaffolds.

## Discussion

Development of liver grafts using traditional tissue engineering approaches has been hampered by a lack of oxygen and nutrient diffusion, which limits the thickness of the cell scaffolds to sheet-like structures^18^. Perfusion decellularization allows liver tissue regeneration of an intact liver structure at a clinically relevant scale by meeting metabolic demands via intact vasculature. In the foreseeable future, the optimal approach for producing whole-liver grafts is to repopulate the decellularized matrix scaffold with parenchymal and nonparenchymal cells and conditioning the graft *in vitro* through perfusion with nutrients^19^. The repopulated graft can be heterotopically transplanted as an auxiliary support to a dysfunctional organ^11^.

From our own perspective, human livers will be the preferred source of organs for clinical trials of decellularization techniques; however, if this process is used for commercial-scale production, pig livers, which are easy to obtain and their quality is easy to ensure, could be preferable in order to reduce costs and for ease of standardization and quality assurance. In this study, SDS-based perfusion decellularization was applied to whole porcine livers to generate xenoantigen-reduced and biocompatible organ scaffolds that preserve the ECM composition and architecture, at a clinically relevant scale.

Although orthotopic or heterotopic transplantation of these acellular scaffolds is possible, the scaffolds are thrombogenic even with anti-coagulation^10^,^20^. The prothrombotic properties of collagen are a major drawback in applications for DLSs. Endothelial coverage of the lumen of the vasculature is essential to prevent thrombosis and to provide proper vascular function. Re-endothelialization of the vascular network of the DLSs would be a major step on development of DLSs^21^ .However, in practice, endothelial cells must be delivered in a manner that appropriately localizes them to the vascular conduit surfaces and the hepatic sinusoid cavity, and achieving near-perfect endothelial cell coverage of the vasculature in the scaffold is challenging. For this reason, most attempts at transplantation of whole-liver scaffolds have been limited to only a few hours^11^. In a previous study, our group developed a promising solution to deposit heparin layers on the DLSs, rendering the whole-liver graft blood-compatible^8^. In the present study, the characteristics of the porcine DLSs immobilized with heparin via LBL, MPA, and EPA were estimated. The non-heparinized counterpart was used as control. We build on our previous work to show that perfusion-decellularized DLSs can be heparinized effectively, reducing the thrombogenicity of the scaffolds.

In order to scale-up the method of heparin immobilization adapted from previous thin tissues^13^,^22^ and rodent studies^8^ to porcine whole-liver scaffolds, the solution perfusion was employed instead of immersion. According to the literatures, the final concentrations of heparin solution were 2 g/L^8^, 4.2 g/L^13^ and 3.3 g/L^22^ for LBL, MPA and EPA, respectively. However, the concentration of 4.2 g/L heparin and N-(3-dimethylaminoporpyl)-N′-ethylcarbodiimide (heparin-EDC) solution for MPA technique is suspension that cannot be used for perfusion until the concentration was decreased to 1 g/L when it become clear solution. In order to make the three methods be comparable, the same heparin concentrations (1, 0.5 or 0.25 g/L) were chosen for MPA, LBL and EPA techniques. Although reducing the heparin concentration in the solutions, homogeneous and complete heparin immobilization via each technique quantified by heparin content criterion and TB staining in all the different segments of the liver. It appeared that EPA is more efficiency of heparin immobilization on DLSs than LBL or MPA method (Table 1). In addition, 2 g/L heparin solution for LBL^8^ and 3.3 g/L heparin solution for EPA^22^ were tested on DLSs, the heparin concentrations immobilization on DLSs were reached 12.82 ± 1.13 μg/mg dry weight and 20.67 ± 0.25 μg/mg dry weight (n = 3), respectively. Obviously, LBL modification process is the most complicated and tedious. Furthermore, the heparin contents can be conveniently adjusted through heparin solution perfusion time.

Heparin is an anionic linear polysaccharide consisting of two repeating disaccharide units: D-glucosamine-L-iduronic acid and D-glucosamine-L-glucuronic acid^23^. Heparinized biological tissues have been used to manufacture blood-contacting prostheses^12^. The heparinization method applied for producing the /h-LBL scaffold was developed by our group. Positively charged polymer PDADMAC was used as a “bridge” to capture the negatively charged heparin and to adsorb to the collagen surfaces via an LBL self-assembly technique. The heparin can be covalently immobilized to collagenous scaffold by activating its carboxyl groups using carbodiimide. The immobilized heparin on DLSs can be MPA because of the many free carboxyl groups in heparin for /h-MPA. The immobilization of nitrite-depolymerized heparin can be covalent EPA on the DLSs as /h-EPA. Nitrous acid can make the heparin partially depolymerize and yield a terminal free-aldehyde functional group on the anhydro mannose unit^14^. The partially depolymerized heparin molecules were linked to the free amino groups on the scaffold via EPA using this terminal free-aldehyde functional group^14^. Heparin may have been sustained in /h-MPA and /h-EPA more than in /h-LBL because the heparin covalently immobilized on the scaffold interacted more strongly than the heparin electrostatically immobilized on the scaffold. The above results reveal that heparin can release slowly from the scaffolds, and that the scaffolds therefore possess excellent anticoagulation capacity (Fig. 4).

**Figure 1:**
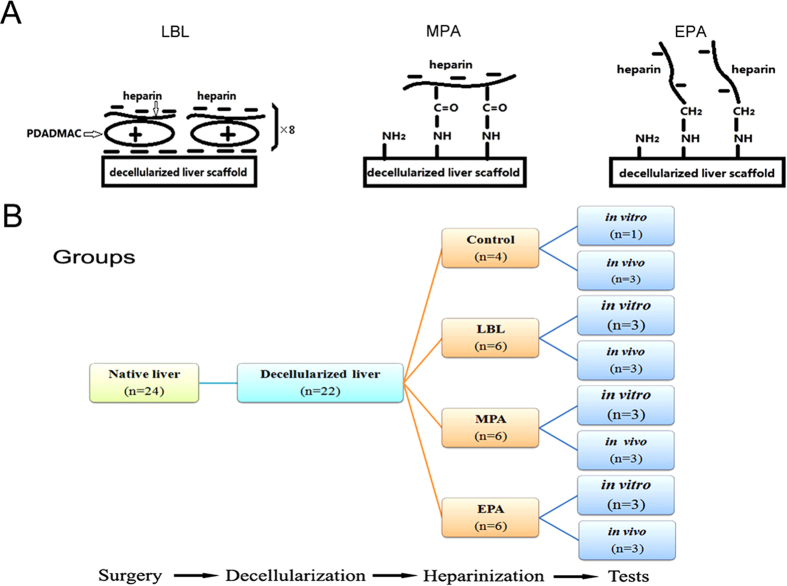
Experiment design. (**A**)Schematic of the structures of heparin immobilized on decellularized liver scaffolds (DLSs). (left) the DLS immobilized with heparin using the layer-by-layer (LBL) technique; (middle) the DLS covalently immobilized with heparin using the multi-point attachment (MPA) technique; (right) the DLS covalently immobilized with heparin using the end-point attachment (EPA) technique. (**B**) Flow chart of the anticoagulation of DLSs through heparin immobilization experiment. The 22 whole livers were perfusion decellularized successfully out of 24 isolated livers from mini-pigs (weight 10–15 kg). The 18 DLSs were divided into 3 groups for LBL, MPA or EPA heparin immobilization, respectively. In each group, half of heparinized DLSs (n = 3) were transplanted into recipient mini-pigs (weight 23–25 kg), and the other half (n = 3) was used for examination *in vitro*. Non-heparin treated DLSs were used as control.

**Figure 2:**
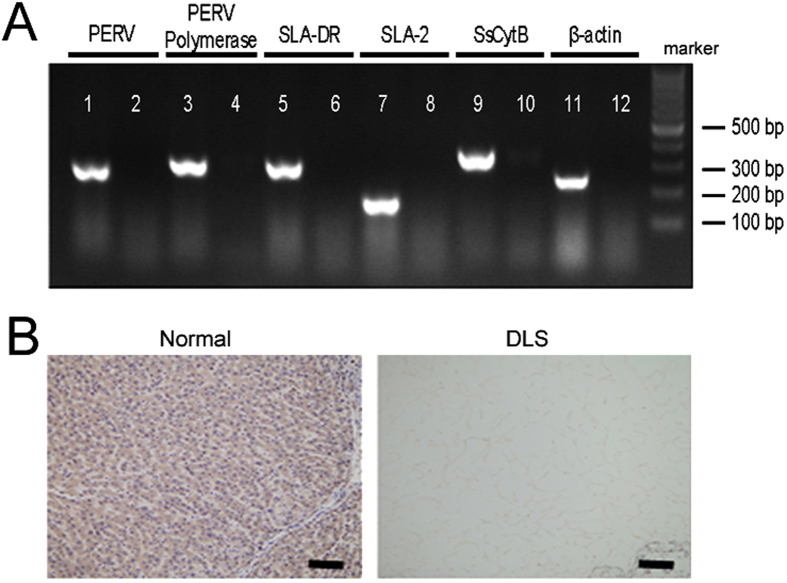
Xenogeneous antigens reduction of DLSs. (**A**) The PCR semi-quantification for porcine endogenous retrovirus (PERV), PERV polymerase (PERV Poly), swine leukocyte antigen DR alpha (SLA-DRA), swine leukocyte antigen 2 (SLA-2), sus scrofa cytochrome b (SsCytb) and porcine beta actin (β-actin) in the DLSs. All the DNA sequences encoding potentially pathogenic or immunogenic antigens were not detected after decellularization process. (**B**) Immunohistochemistry staining of normal liver tissue and DLSs for α-Gal. Scale bars, 100 μm.

PF4 has been reported to play a vital role in platelet aggregation^24^, then, PF4 was used to detect platelet activation by biomedical materials^17^. Platelet adhesion on the scaffolds is of primary importance for blood compatibility. Before platelet adhesion, plasma proteins are quickly adsorbed on the scaffold surface, and the adsorbed proteins strongly affect subsequently blood-material surface interactions. Fibrinogen appears to be an important component of this adsorption process. This plasma protein is known to be capable of mediating platelet activation, aggregation and adhesion via direct interactions with platelet receptors such as GP IIb/IIIa^25^. The ability of surface-immobilized heparin on collagenous tissue to prevent thrombus formation through disturbed fibrinogen adsorption has been reported in the literature^12^,^22^. Furthermore, in the /h-EPA tissue, heparin molecules were covalently immobilized on the tissue surface like bristles (Fig. 1A-right). The fibrinogen molecules that were adsorbed on swinging bristles, rather than on the tissue surface, may then be easily washed away during the deionized water rinsing procedure prior to desorption. Field counts of scanning electron images showed greater adhesion of platelets on the surfaces of the nonheparinized tissues than on their heparinized counterparts (Fig. 6A).

The most direct test of the anti-thrombotic benefit of these techniques is perfusion of the whole organ to whole blood and quantification of thrombus formed in the whole organ. With or without heparin treated DLSs were transplanted into the porcine receipt with successful reperfusion of the scaffolds, which acquired a liver-like macroscopic appearance. Notably, no extravasation occurred either during the operative period, which suggests that under normal circumstances vessel watertight sealing depends mainly on the integrity of the scaffold’s bare vasculature rather than the endothelium. Despite the pre-administration of anticoagulation prophylaxis in DLSs, the whole vascular network of control DLSs was occluded by massive clots within 20 min after perfusion. Heparinized DLSs remained blood flow throughout the whole intraoperative observation time of at least 1 hour. However, the blood flow rate decreased significantly from the inflow to the outflow of heparinized DLSs, due to the complete absence of hepatocytes and endothelium, massive blood entered and trapped in the original liver parenchyma and hepatic sinus areas.

These results indicate that DLSs pretreated with heparin can decrease thrombus formation and increase the graft patency. However, engineering an intact, functional whole-liver graft will require recellularizing liver parenchyma with hepatocytes and covering the vascular bed with endothelial cells to restore the unique, functional characteristics of the hepatic microvascular circulation and to prevent coagulation^20^,^26^. Heparin treated DLSs did not compromise their cytocompatibility, which could be reendothelialized with HUVECs and recellularized with rat primary hepatocytes as non-treated DLSs^9^,^19^,^26^.

Prevention of blood coagulation is difficult to achieve, and failure to do so hinders long-term *in vivo* testing of recellularized liver grafts. This concern may be overcome by immobilizing heparin on the DLSs, as shown in this study. The three types of heparinized DLSs showed different efficiency of heparin immobilization. The heparinized scaffolds showed improved anticoagulation and cytocompatibility compared to the control scaffold both *in vitro* and *in vivo* test. The /h-EPA scaffold showed a dramatic improvement in hemocompatibility compared to the other scaffolds. Whole-organ tissue engineering is complex and involves many technically challenging steps that must be performed successfully in series. Although heparinization of the grafts is a major improvement, the efficacy and safety of this technique still require further longtime *in vivo* testing.

## Conclusion

This report provides an efficient method for immobilization of heparin on DLSs, which could provide a solution to the problem of anticoagulation ability in liver engineering. The data presented here show some advantages in the use of the EPA heparinization method to improve blood and cell compatibility of DLSs. These hemocompatibility improvement studies are a first step toward generating a transplantable functional liver graft using DLSs.

## Materials and methods

### Animals

24 livers were isolated from male Bama miniature pigs weighing 10–15 kg for perfusion decellularization. 12 Bama miniature pigs weighing 23–25 kg were employed as DLSs transplantation receipts. A 220 g male Sprague–Dawley rat was used for hepatocyte isolation. All animals were obtained from the Animal Experiment Center of Sichuan University (Chengdu, China). All animal experiments were performed in accordance with the Animal Welfare Act and approved by the institutional animal care and use committee at Sichuan University.

### Perfusion decellularization of porcine liver

The surgeries were performed under ketamine (6 mg/kg body weight, administered IP, Kelun, Chengdu, China) and xylazine (10 mg/kg IP, Kelun) anesthesia. Under deep anesthesia, a laparotomy was performed and the liver was exposed. After systemic heparinization through the inferior vena cava, the hepatogastric ligament was carefully dissected. The proximal PV was catheterized. The hepatic artery and common bile duct were ligated and transected. All perihepatic ligaments were severed. Simultaneously, the liver was slowly perfused with 2 L deionized water containing 0.1% EDTA (Kelun) through a cannula in the PV, and the SHIVC was transected, allowing outflow of the perfusate. Following blanching, the liver was stored at −80 °C overnight. The liver was perfused with 1% Triton X-100 (Amresco, Solon, OH, USA) for 3 h and then by 1% SDS (Promega, San Luis Obispo, CA, USA) in deionized water at a rate of 200 mL/min for 6 h after thawing. This was followed by 3 h of perfusion with 1% Triton X-100 to remove residual SDS. Subsequently, the liver was washed with 20 L of distilled water to remove residual detergent, followed by infusion of 40 L of phosphate-buffered saline (PBS) at 200 mL/min.

### Polymerase chain reaction (PCR) analysis

Genomic DNA content in native and decellularized livers was normalized to the initial weight of the sample. The sequences of primers were listed in Table 2. PCR products were loaded on 2% agarose gel and visualized by the GoldView nucleic acid staining solution.

### Perfusion heparin treatment

#### LBL technique

The /h-LBL scaffolds were prepared using the LBL self-assembly technique for heparin immobilization, as previously described^8^. The polyelectrolyte polydiallyldimethylammonium chloride (PDADMAC, Mw = 100–200 kDa, positively charged, Sigma, USA), which is oppositely charged to heparin (Kelong, Chengdu, China), was used. Typical adsorption conditions were used to form multiple layers on the surfaces of DLS. Following a 30-min perfusion of PDADMAC or heparin (1 g/L of PDADMAC or 2, 1, 0.5 or 0.25 g/L heparin in PBS) for each step at 100 mL/min through PV. After each perfusion, the DLS were washed via PBS perfusion for 10 min to eliminate residual polyelectrolytes. The eight-bilayer (PDADMAC/heparin)_8_ on top was deposited onto the internal matrix surface of the DLS.

#### MPA technique

The method modified from Wang *et al.*^13^ was used to immobilize heparin on DLS via MPA to prepare the /h-MPA scaffolds. The DLS were treated for heparin immobilization by PV perfusion 1 mol/L hydroxylamine sulfate salt (Sigma) at 100 mL/min for 12 h, followed by 3 L distilled water perfusion at 200 mL/min to rinse, then heparin-EDC solution (2 g EDC (Sigma) +1, 0.5 or 0.25 g heparin sodium salt (Kelong) +1 L 0.05 M HCl, pH 1.5, prepared in our laboratory) were cycled at 100 mL/min for 4 h at 37 °C.

#### EPA technique

Heparin was immobilized on the DLS via EPA according to the method modified from Larm et al.^14^. Firstly, sodium heparin was dissolved in distilled water (1 g/300 mL) at approximately 0 °C. The dissolved heparin was partially depolymerized by adding sodium nitrite (10 mg) to the solution (pH 2.7, adjusted with 1N HCl) and then stirred at 0 °C for 2 h. Subsequently, the pH of the solution was adjusted to 7.0 with 1N NaOH. The solution containing partially depolymerized heparin (Diluted to the concentration of heparin 3.3, 1, 0.5 or 0.25 g/L) with NaBH_3_CN (0.01 mg/mL) and NaCl (0.15 M) at pH3.5 were perfused into DLSs through PV at 37 °C, and then recycled at 100 mL/min for 4 h. Nitrite-depolymerized heparin can be immobilized via covalent end-point attachment to the DLS^14^.

**Figure 3:**
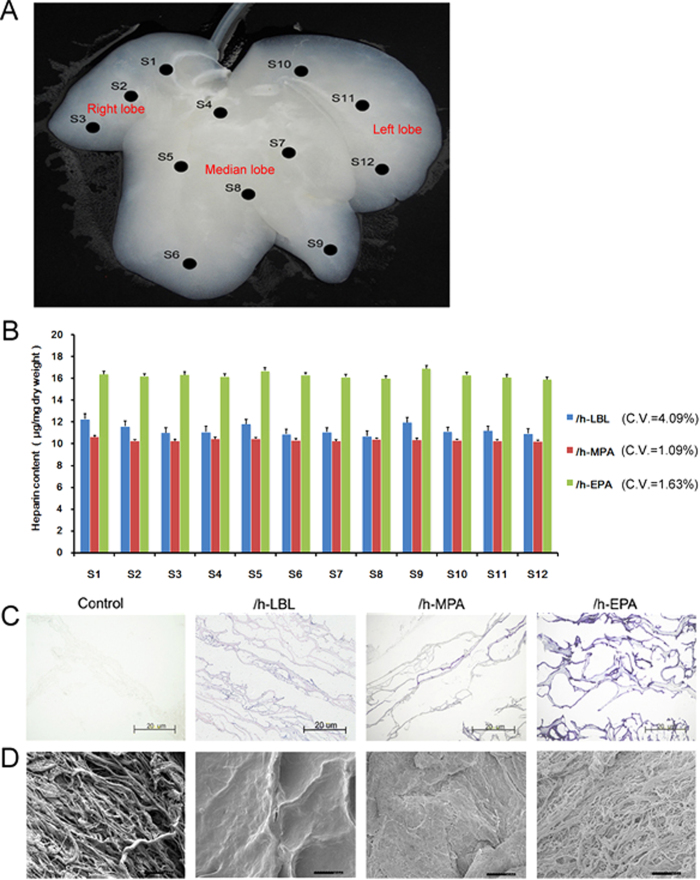
Whole-organ decellularized porcine liver homogeneous heparin immobilization. (**A**) Samples were taken from 12 different sites (S1 to S12) of DLS different lobes. (**B**) The heparin content results of S1 to S12 from each liver with different heparin treatments, the coefficients of variations (CV %) for three methods were 4.09% (LBL), 1.09% (MPA) and 1.63% (EPA) respectively; values are expressed as means ± SD, n = 3. (**C**) TB staining of heparin immobilized on DLSs, blue staining is positive for heparin immobilization. (**D**) SEM micrographs of heparin immobilized on DLSs. (**C**) and (**D**) from left to right: control, /h-LBL, /h-MPA, and /h-EPA. Scale bars: 20 μm.

**Figure 4:**
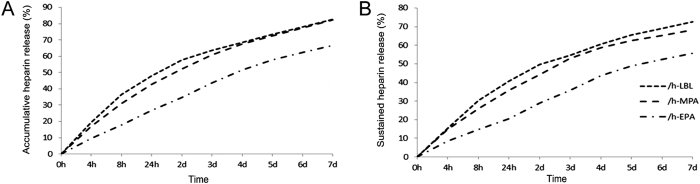
Release curves of heparinized DLSs. (**A**) Accumulative heparin release curve and (**B**) sustained heparin release curve of heparinized DLSs over 7 days; values are expressed as means, n = 3.

**Figure 5:**
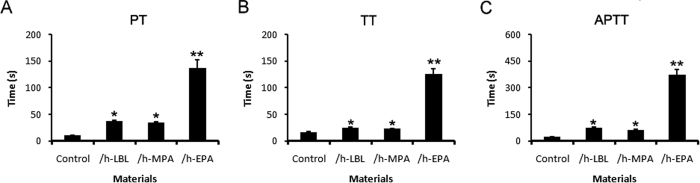
Coagulation time of different heparinized DLSs. (**A**) prothrombin time (PT), (**B**) thrombin time (TT), and (**C**) activated partial thromboplastin time (APTT) values of control and alternately heparinized DLSs; values are expressed as means ± SD, n = 3. *p < 0.05, **p < 0.001 vs. the control group.

**Figure 6:**
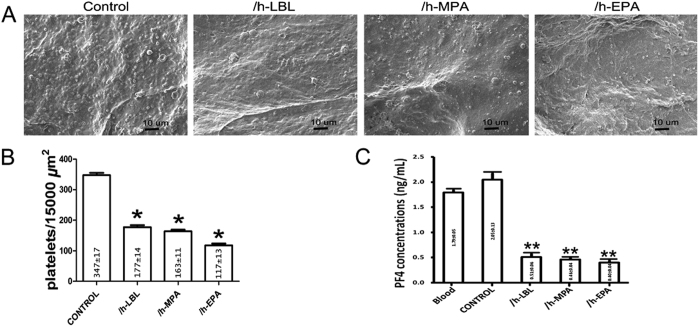
Platelet adhesion and activation of different heparinized DLSs. (**A**) SEM micrographs of platelets adhered to different scaffolds (from left to right: control, /h-LBL, /h-MPA, and /h-EPA). Scale bars: 20 μm. (**B**) Amount of platelets adhered on each test DLSs. (**C**) Generated concentrations of platelet factor 4 (PF4) of these DLSs with whole blood, which can act as an index for platelet activation. Values are expressed as means ± SD, n = 3, *p < 0.05, **p < 0.001 vs. the control group.

**Figure 7:**
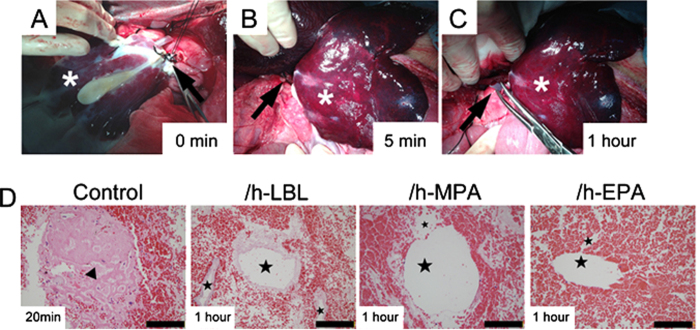
Transplantation of the DLSs into the infrahepatic space of a live pig. (**A**) The recipient’s renal vein (RV) and DLS’s portal vein (PV) (arrow) were used to perform end-to-end anastomosis between the recipient and the DLSs. The blood filled DLS immediately after reperfusion. (**B**) Blood perfused homogenously throughout the DLS within 5 min. The suprahepatic inferior vena cava (SHIVC) of DLS has been reconnected to the recipient’s splenic vein (SV) (arrow). (**C**) The perfused DLS after 1 h of perfusion. The blood flow out from SHIVC (arrow) of DLS. * EPA heparin-treated DLS. (**D**) H&E staining of the explanted DLSs shows vascular structures of control group DLSs are occluded by thrombosis (triangle) after 20 min of perfusion, whereas others are open with red blood cells (star) after 1 h of perfusion, from left to right: control, /h-LBL, /h-MPA, and /h-EPA. 3 DLSs implantation for each group. Scale bars: 50 μm.

**Figure 8:**
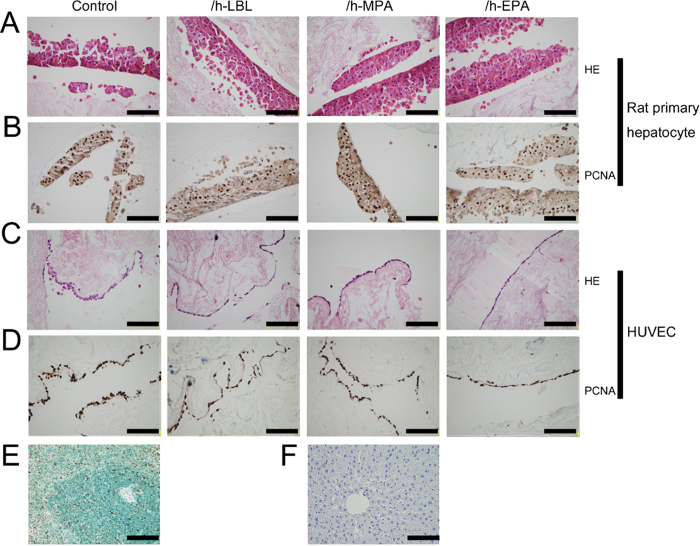
Cells seeded on the various heparinized DLSs for 3 days. (**A**) H&E staining of rat primary hepatocytes. (**B**) Immunohistochemical analysis of rat primary hepatocytes proliferation stained for proliferating cell nuclear antigen (PCNA), approximately 80% of cells stained positive (brown nuclei) in each group. (**C**) H&E staining of human umbilical vein endothelial cells (HUVECs). (**D**) Immunohistochemical analysis of HUVECs proliferation stained for PCNA, approximately 100% of cells stained positive (brown nuclei) in each group. (**E**) Immunohistochemical stained rat spleen tissue for PCNA as positive staining control, brown nuclear is positive stained. (**F**) Immunohistochemical stained rat liver tissue for PCNA as negative staining control. From left to right: control, /h-LBL, /h-MPA, and /h-EPA. Scale bars: 50 μm.

Each heparin immobilized DLSs were sampled from 12 sites showed in Fig. 3A and cut into 300-μm thick sections with a diameter of 1 cm and used in the following *in vitro* experiments.

### TB staining

The non-heparinized and heparinized DLSs were stained with 1% (w/v) aqueous TB, as previously described^27^.

### Heparin contents test

The quantity of heparin immobilized on the three DLSs was determined as Smith et al.^28^ described.

### Heparin release rate test

To determine heparin release, the three different types of heparinized DLSs were each fixed at the bottom a well in a 24-well plate and immersed in PBS solution (2.5 mL, pH 7.4) for 7 days. To determine the accumulative release, solution (2.5 mL) was periodically collected from each well and replaced with fresh solution, and the heparin content released each day was recorded. To determine the sustained release, the solution was collected without replacement until the final time point. The heparin concentration in PBS solution was determined as described^28^.

### *In vitro* coagulation time tests

To evaluate the antithrombogenicity of the heparinized DLSs, PT, APTT and TT were measured by a semiautomatic blood coagulation analyzer CA-50 (Sysmex Corporation, Kobe, Japan) as described^29^,^30^.

### Platelet adhesion

The experimental procedure employed in the platelet adhesion study for DLSs was reported by Ko *et al.*^31^. Scanning electron microscopy (SEM, JSM-7500F, JEOL, Japan) was performed to count the total number of adhered platelets on DLSs^22^.

### Platelet activation

Platelet activation (PF4) was evaluated via commercial enzyme-linked immunosorbent assay (ELISA) kit (E-(Hu)(11)-04605, Yanyu, Shanghai, China). Whole blood was incubated with the heparinized or control DLSs for 2 h and centrifuged at 1000 × g (4 °C) for 15 min to obtain plasma. Then detections were conducted according to the manufacturer’s instruction.

### *In vivo* implantation of heparinized DLSs

To examine the anti-thrombotic ability of the heparinized DLSs under whole blood perfusion, we performed an auxiliary transplantation of the DLS into a recipient porcine model. Considering the blood volume of recipient, the right lobe and left lobe were removed, the median lobe was preserved with PV and SHIVC. Before implanted, 100 mL saline with 25 IU/mL heparin was injected into DLS through PV. The DLSs was implanted in the infrahepatic space by using the recipient left renal vein (RV) and splenic vein (SV) as an inflow and outflow, respectively. After positioning of a clamp on the recipient’s RV and SV, scaffold’s PV and SHIVC were anastomosed end to end using silicone cuff, respectively. After reperfusion, scaffolds were monitored for 1 hour before retrieved for pathology studies.

### Cell culture

Hepatocytes were isolated from a 220 g male Sprague–Dawley rat by a two-step perfusion method as our previously described^32^. All harvests yielded hepatocytes with viability exceeding 95% by trypan-blue dye exclusion. HUVECs were cultured in DMEM medium supplemented with 10% fetal bovine serum (FBS, Hyclone, America). To evaluate the cytocompatibility of the scaffolds, primary hepatocytes or HUVECs (2.5 × 10^4^ cells/cm^2^) were cultured inside the control, /h-LBL, /h-MPA, and /h-EPA scaffolds (n = 3) in 24-well culture plates, respectively. The viable cells cultured on each test specimen were examined by H&E staining at 3 days after cell seeding. The proliferation of the cells was evaluated using the PCNA (1:2000, rabbit polyclonal IgG, ab18197; abcam, Shanghai, China) immunohistochemistry staining.

### Statistical analysis

The coagulation time tests of the heparin-treated and non-heparin-treated scaffolds were compared using a χ^2^test. For comparison of heparin contents and cell number on the scaffolds, a one-way analysis of variance (ANOVA), followed by a Student-Newman-Keuls test for variability, was performed. The data are presented as mean ± SD, and *p* < 0.05 was considered statistically significant.

## Additional Information

**How to cite this article**: Bao, J. *et al.* Hemocompatibility improvement of perfusion-decellularized clinical-scale liver scaffold through heparin immobilization. *Sci. Rep.* **5**, 10756; doi: 10.1038/srep10756 (2015).

## Supplementary Material

Supplementary Information

## Figures and Tables

**Table 1:** Heparin contents of different heparinized DLSs (μg/mg dry weight, n = 3, ± SD)

	**Concentrations of heparin in solutions (g/L)**		
**Test samples**	**1**	**0.5**	**0.25**
/h-LBL	11.17 ±0.05	4.26 ±0.61	0.81 ±0.42
/h-MPA	10.30 ±0.06	6.21 ±0.18	3.09 ±0.28
/h-EPA	16.00 ±0.02	9.08 ±0.14	2.25 ±0.21

**Table 2:** PCR primer sequences and product length.

**Gene**	**Abbr.**	**Assession No.**	**Forward primers**	**Reverse primers**	**Size of product**
Porcine endogenous retrovirus	PERV	NC_003059	5′-CTACCCCGAGATTGAGGAGC-3′	5′-GGGGGATGGTTAGTTTTCCA-3′	297 bp
PERV Polymerase	PERV Poly	EU_789636	5′-CTACAACCATTAGGAAAACTAAAAG-3′	5′-AACCAGGACTGTATATCTTGATCAG-3′	326 bp
Swine leukocyte antigen DR alpha	SLA-DRA	NC_010443	5′-CGAGAAGAGGTGGCAAGACA-3′	5′-GTCCTGGAGGATTCCCTTGA-3′	343 bp
Swine leukocyte antigen 2	SLA-2	NC_010449	5′-GTCACCTTGAGGTGCTGGG-3′	5′-TGGCAGGTGTAGCTCTGCTC-3′	185 bp
Sus scrofa cytochrome b	SsCytB	NC_000845	5′-CATTGGAGTAGTCCTACTATTTACCG-3′	5′-GTAGGATTAGTATTATAAATAAGGCTCCT-3′	377 bp
Porcine beta actin	β-actin	NC_010454	5′-TGTCATGGACTCTGGGGATG-3′	5′-GGGCAGCTCGTAGCTCTTCT-3′	277 bp

## References

[b1] Badylak S. F. The extracellular matrix as a biologic scaffold material. Biomaterials 28, 3587–3593 (2007). 17524477 10.1016/j.biomaterials.2007.04.043

[b2] Ajalloueian F., Zeiai S., Rojas R., Fossum M. & Hilborn J. One-stage tissue engineering of bladder wall patches for an easy-to-use approach at the surgical table. Tissue Eng Part C Methods 19, 688–696 (2013). 23327166 10.1089/ten.TEC.2012.0633

[b3] Guan Y. *et al.* Tissue engineering of urethra using human vascular endothelial growth factor gene-modified bladder urothelial cells. Artif Organs 32, 91–99 (2008). 18005271 10.1111/j.1525-1594.2007.00502.x

[b4] Schechner J. S. *et al.* Engraftment of a vascularized human skin equivalent. FASEB J 17, 2250–2256 (2003). 14656987 10.1096/fj.03-0257com

[b5] Gonfiotti A. *et al.* The first tissue-engineered airway transplantation: 5-year follow-up results. Lancet 383, 238–244 (2014). 24161821 10.1016/S0140-6736(13)62033-4

[b6] Dahl S. L., Koh J., Prabhakar V. & Niklason L. E. Decellularized native and engineered arterial scaffolds for transplantation. Cell Transplant 12, 659–666 (2003). 14579934

[b7] Nieponice A., Gilbert T. W. & Badylak S. F. Reinforcement of esophageal anastomoses with an extracellular matrix scaffold in a canine model. Ann Thorac Surg 82, 2050–2058 (2006). 17126109 10.1016/j.athoracsur.2006.06.036

[b8] Bao J. *et al.* Construction of a portal implantable functional tissue-engineered liver using perfusion-decellularized matrix and hepatocytes in rats. Cell Transplant 20, 753–766 (2011). 21054928 10.3727/096368910X536572

[b9] Yagi H. *et al.* Human-scale whole-organ bioengineering for liver transplantation: a regenerative medicine approach. Cell Transplant 22, 231–242 (2013). 22943797 10.3727/096368912X654939 PMC3682787

[b10] Orlando G. *et al.* Production and implantation of renal extracellular matrix scaffolds from porcine kidneys as a platform for renal bioengineering investigations. Ann Surg 256, 363–370 (2012). 22691371 10.1097/SLA.0b013e31825a02ab

[b11] Uygun B. E., Yarmush M. L. & Uygun K. Application of whole-organ tissue engineering in hepatology. Nat Rev Gastroenterol Hepatol 9, 738–744 (2012). 22890112 10.1038/nrgastro.2012.140 PMC3732057

[b12] Noishiki Y. & Miyata T. A simple method to heparinize biological materials. J Biomed Mater Res 20, 337–346 (1986). 3957968 10.1002/jbm.820200306

[b13] Wang X. N., Chen C. Z., Yang M. & Gu Y. J. Implantation of decellularized small-caliber vascular xenografts with and without surface heparin treatment. Artif Organs 31, 99–104 (2007). 17298398 10.1111/j.1525-1594.2007.00348.x

[b14] Larm O., Larsson R. & Olsson P. A new non-thrombogenic surface prepared by selective covalent binding of heparin via a modified reducing terminal residue. Biomater Med Devices Artif Organs 11, 161–173 (1983). 6667322 10.3109/10731198309118804

[b15] Ma X., Cao C. & Zhu H. The biocompatibility of silk fibroin films containing sulfonated silk fibroin. J Biomed Mater Res B Appl Biomater 78, 89–96 (2006). 16292767 10.1002/jbm.b.30466

[b16] Gorbet M. B. & Sefton M. V. Biomaterial-associated thrombosis: roles of coagulation factors, complement, platelets and leukocytes. Biomaterials 25, 5681–5703 (2004). 15147815 10.1016/j.biomaterials.2004.01.023

[b17] Deng J. *et al.* Heparin-Mimicking Multilayer Coating on Polymeric Membrane via LbL Assembly of Cyclodextrin-Based Supramolecules. ACS Appl Mater Interfaces 6, 21603–21614 (2014). 25375347 10.1021/am506249r

[b18] Ohashi K. *et al.* Engineering functional two- and three-dimensional liver systems *in vivo* using hepatic tissue sheets. Nat Med 13, 880–885 (2007). 17572687 10.1038/nm1576

[b19] Sabetkish S. *et al.* Whole-organ tissue engineering: Decellularization and recellularization of three-dimensional matrix liver scaffolds. J Biomed Mater Res A 10.1002/jbm.a.35291 (2014). 10.1002/jbm.a.35291 25045886

[b20] Robertson M. J., Dries-Devlin J. L., Kren S. M., Burchfield J. S. & Taylor D. A. Optimizing recellularization of whole decellularized heart extracellular matrix. PLoS One 9, e90406 (2014). 24587354 10.1371/journal.pone.0090406 PMC3937369

[b21] Baptista P. M. *et al.* The use of whole organ decellularization for the generation of a vascularized liver organoid. Hepatology 53, 604–617 (2011). 21274881 10.1002/hep.24067

[b22] Tsai C. C., Chang Y., Sung H. W., Hsu J. C. & Chen C. N. Effects of heparin immobilization on the surface characteristics of a biological tissue fixed with a naturally occurring crosslinking agent (genipin): an *in vitro* study. Biomaterials 22, 523–533 (2001). 11219715 10.1016/s0142-9612(00)00206-4

[b23] Amiji M. & Park K. Surface modification of polymeric biomaterials with poly(ethylene oxide), albumin, and heparin for reduced thrombogenicity. J Biomater Sci Polym Ed 4, 217–234 (1993). 8476792 10.1163/156856293x00537

[b24] Niewiarowski S. & Thomas D. P. Platelet factor 4 and adenosine diphosphate release during human platelet aggregation. Nature 222, 1269–1270 (1969). 5789664 10.1038/2221269a0

[b25] Horbett T. A. Chapter 13 Principles underlying the role of adsorbed plasma proteins in blood interactions with foreign materials. Cardiovasc Pathol 2, 137–148 (1993).25990608

[b26] Barakat O. *et al.* Use of decellularized porcine liver for engineering humanized liver organ. J Surg Res 173, e11–25 (2012). 22099595 10.1016/j.jss.2011.09.033

[b27] Krzanowska H. Toluidine blue staining reveals changes in chromatin stabilization of mouse spermatozoa during epididymal maturation and penetration of ova. J Reprod Fertil 64, 97–101 (1982). 6172584 10.1530/jrf.0.0640097

[b28] Smith P. K., Mallia A. K. & Hermanson G. T. Colorimetric method for the assay of heparin content in immobilized heparin preparations. Anal Biochem 109, 466–473 (1980). 7224172 10.1016/0003-2697(80)90679-x

[b29] Zhang W. *et al.* Effect of chitosan and carboxymethyl chitosan on fibrinogen structure and blood coagulation. J Biomater Sci Polym Ed 24, 1549–1563 (2013). 23848448 10.1080/09205063.2013.777229

[b30] Xiang T. *et al.* From commodity polymers to functional polymers. Sci Rep 4, 4604 (2014). 24710333 10.1038/srep04604 PMC3978497

[b31] Ko T. M., Lin J. C. & Cooper S. L. Surface characterization and platelet adhesion studies of plasma-sulphonated polyethylene. Biomaterials 14, 657–664 (1993). 8399962 10.1016/0142-9612(93)90064-9

[b32] Bao J. *et al.* Serum-free medium and mesenchymal stromal cells enhance functionality and stabilize integrity of rat hepatocyte spheroids. Cell Transplant 22, 299–308 (2013). 23006214 10.3727/096368912X656054 PMC3781336

