# Supplementary material for: Hemocompatibility improvement of perfusion-decellularized clinical-scale liver scaffold through heparin immobilization
Source: Sci Rep. 2015 Jun 1;5:10756. doi: 10.1038/srep10756 (PMC5377232; doi:10.1038/srep10756)
Supplement: Supplementary Information [file srep10756-s1.doc]

**Hemocompatibility improvement of perfusion-decellularized clinical-scale liver scaffold through heparin immobilization**

Ji Bao^1,2,3^, Qiong Wu^1,3^, Jiu Sun^4^, Yongjie Zhou^1,3^, Yujia Wang^1,3^, Xin Jiang^5^, Li Li^1,3^, Yujun Shi^1,3^, Hong Bu^1,2,3^*

1. Laboratory of Pathology, West China Hospital, Sichuan University, Chengdu, 610041, China

2. Department of Pathology, West China Hospital, Sichuan University, Chengdu, 610041, China

3. Key Laboratory of Transplant Engineering and Immunology, Ministry of Health, West China Hospital, Sichuan University, Chengdu, 610041, China

4. Department of General Surgery, The first people’s hospital of Yibin, Yibin, 644000, China

5. College of Polymer Science and Engineering, Sichuan University, Chengdu, 610041, China

* Corresponding author. Tel: +86-28-85164030; Fax: +86-28-85164033.

E-mail address: [hongbu@scu.edu.cn](mailto:hongbu@scu.edu.cn) (H. Bu)

**Supplementary Materials**

**Supplementary figures**


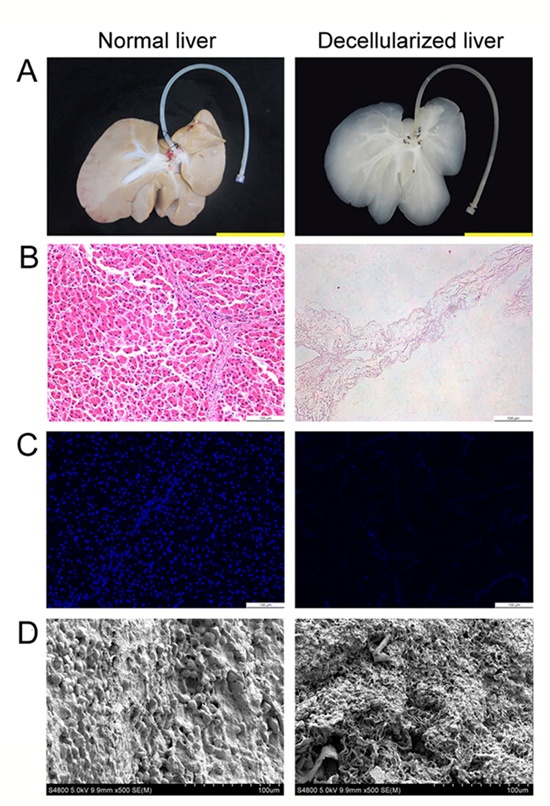


Figure-S1. Perfusion decellularization of whole porcine livers. Representative images for (A) macrograph, (B) hematoxylin and eosin (H&E) staining, (C) DAPI staining and (D) SEM analysis of ultrastructure of normal (left) and decellularized (right) liver. Scale bars: (A) 10cm, (B, C) 100 µm, and (D) 100 µm.


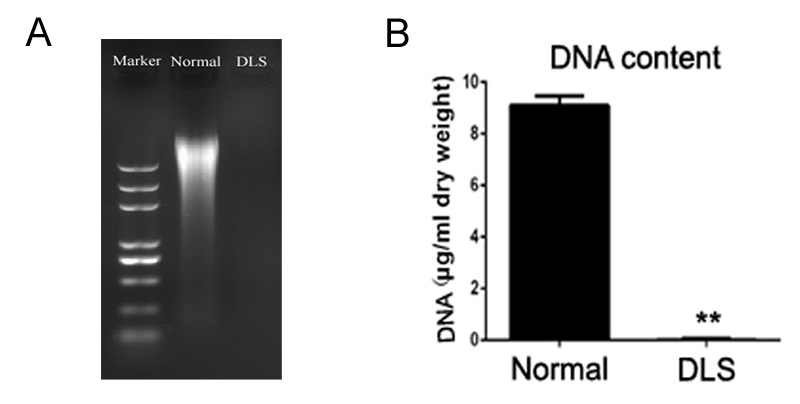


Figure-S2. Quantification of residual DNA in decellularized liver scaffold.

(A) Agarose gel electrophoresis of DNA extracted from the normal and DLS. (B) DNA content in normal liver and DLS was normalized to the initial dry weight of each sample. ***p*< 0.001 vs. normal liver tissue; values are expressed as mean ±SD, n=3. DLS: decellularized liver scaffold.


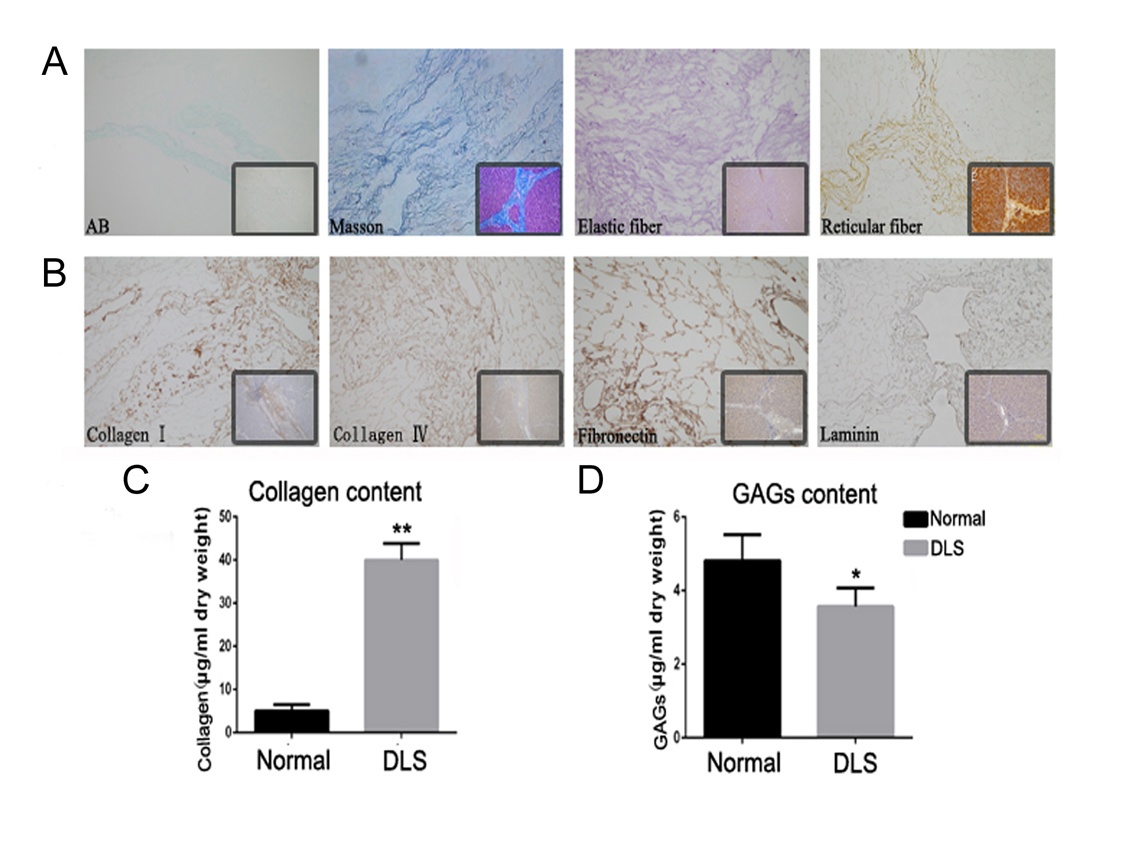


Figure-S3. Retention of EMC components in DLSs. (A) Special staining (left to right: AB staining, Masson’s trichrome staining, elastic fiber staining, and reticular fiber staining) and (B) immunohistochemical staining (left to right: Collagen I, Collagen IV, Fibronectin, and Laminin) of DLS. Normal liver was employed as a control (insert). Original magnification: × 200. The collagen (C), and GAG (D) contents in DLSs. **p*< 0.05 and ** *p*< 0.001 vs. normal liver tissue.

**Supplementary tables**

Table S1. Recalcification time of different heparinized DLSs （n = 3, ± SD）

| Test samples |  | Recalcification time (s) |  | *p.*value (vs. blank) |  | *p.*value | |
| --- | --- | --- | --- | --- | --- | --- | --- |
| negative control |  | 87.0 ± 7.9 |  |  |  |  |  |
| blank control |  | 113.3 ± 15.0 |  |  |  |  |  |
| /h-LBL |  | 254.0 ± 14.0 |  | 0.0001 |  | 0.003 | (vs. /h-EPA) |
| /h-MPA |  | 206.7 ± 25.2 |  | 0.002 |  | 0.023 | (vs. /h-LBL) |
| /h-EPA |  | 350.3 ± 29.1 |  | 0.0001 |  | 0.001 | (vs. /h-MPA) |

Table S2. Hemolysis ratio of different heparinized DLSs （n = 3, ± SD）

| Test samples |  | Haemolysis ratio（%） |  | *p.*value (vs. control) |  | *p.*value | |
| --- | --- | --- | --- | --- | --- | --- | --- |
| Control |  | 8.69 ± 0.634 | |  |  |  |  |
| /h-LBL |  | 4.63 ± 0.517 | | 0.0005 |  | 0.005 | (vs. /h-EPA) |
| /h-MPA |  | 4.70 ± 0.519 | | 0.0005 |  | 0.438 | (vs. /h-LBL) |
| /h-EPA |  | 2.93 ± 0.382 | | 8.76E-05 |  | 0.004 | (vs. /h-MPA) |

**Supplementary methods**

**Histology**

Normal fresh liver, decellularized liver scaffolds, and recellularized liver scaffolds were fixed in 4% paraformaldehyde at room temperature for 24h. They were dehydrated step-wise using ethanol, immersed in xylene, and embedded in paraffin. The ECM samples were cut into 5-μm sections and stained with hematoxylin and eosin (H&E). Sections were mounted in mounting media containing 4′,6-diamidino-2-phenylindole (DAPI) to confirm the extent of decellularization.

**Measurement of DNA content**

To measure DNA content, we took decellularized livers and a sample of the fresh-frozen liver tissue samples as a control. Briefly, total DNA was isolated from 15 mg of tissue (dry weight) using a commercially available kit (Tiangen Biotech Corporation, Beijing, China). The DNA concentration was estimated at 260nm using a NanoDrop spectrophotometer (ND-2000c; Thermo, USA) and normalized to the initial dry weight of the samples. Total DNA was normalized and equal concentrations were loaded onto a 1.5% agarose gel and electrophoresed with a DNA ladder to assess the DNA fragments.

**Scanning electron microscopy (SEM)**

Following decellularization, heparinization and recellularization, samples were fixed in 2.5% glutaraldehyde for at least 2h at room temperature. Following fixation, samples were briefly rinsed in deionized water, dehydrated via a graded ethanol series, and dried in a critical point dryer (HCP2; Hitachi, Tokyo, Japan). Samples were then sputter-coated with gold prior to SEM imaging. Electron micrographs of liver cross-sections were obtained at 5.0 kV, ×1000 magnification using a Hitachi S-4800 SEM (Tokyo, Japan).

**Evaluation of ECM components**

Sections of decellularized liver samples were stained with Masson’s trichrome stain, alcian blue stain, elastic fiber stain, and reticular fiber stain following standard protocols. To determine whether collagen-I (1:1000, rabbit polyclonal IgG, GTX26308; GeneTex, USA), collagen –IV (1:100, rabbit polyclonal IgG, bs-4595R; Biosson, Beijing, China), laminin (1:1000, rabbit polyclonal IgG, GTX11574; GeneTex，USA), fibronectin (GeneTex, 1:100, rabbit polyclonal IgG, GTX72724; GeneTex, USA) and α-Gal (1:100, rabbit polyclonal IgG, bs-7593R; Biosson, Beijing, China) were retained in the decellularized matrices, the liver ECM samples were sectioned and stained using immunohistochemistry. Briefly, paraffin sections were rehydrated, incubated in antigen retrieval solution, and stained using antibodies to fibronectin, laminin, collagen-I and –IV, and α-Gal. Images of the stained slides were captured using an upright microscope (BX51; Olympus, Tokyo, Japan).

**Collagen assay**

Collagen was quantified using a colorimetric assay to detect hydroxyproline. Native liver and the decellularized liver were cut into small pieces, placed in centrifuge tubes, and subsequently lyophilized. The samples were weighed, incubated with papain (140 µg/mL) at 60°Covernight, hydrolyzed in 6M HCl at 115°C for 18h, and then neutralized, oxidized with chloramine-T, and reacted with *p*-dimethylaminobenzaldehyde. The absorbance at 570 nm was obtained, and a 1:10 w/w ratio of hydroxyproline to collagen was used to calculate the collagen content of the tissue. At least 4 parallel samples were analyzed for native and decellularized samples.

**Glycosaminoglycans (GAGs) assay**

Sulfated GAGs were quantified using the Blyscan GAG assay kit (B1000; Biocolor, Carrickfergus, UK). The samples were lyophilized, weighed, and then incubated with papain (150 µg/mL) at 65°C for 3 h. The supernatants were placed in 1.5-mL tubes. After Blyscan dye reagent was added, the content of the tubes was homogenized for 30 min and centrifuged for 10 min (10,000 ×*g*). The deposits were dissolved with dissociation reagent and absorbance was read at 650 nm. At least 4 samples were analyzed for native and decellularized samples.

**Plasma recalcification time**

The plasma recalcification time was determined as reported previously ^1^ ^2^, with some modifications. Briefly, the scaffolds were placed in uniformly sized glass tubes. The non-heparinized DLSs were used as a blank control, and tubes without scaffolds were used as a negative control. 500 μL of plasma and 200 μL of a 0.025 mol/L aqueous solution of CaCl_2_ were added to each tube. The plasma solution was monitored for clotting by detecting fibrin threads. The first sign of fibrin formation in the solution was recorded as the clotting time. A minimum of six experiments were carried out on each substrate surface, and the mean value is reported as the plasma recalcification time.

**Hemolysis ratio measurement**

Anticoagulant citrate dextrose (ACD) blood was provided by the Laboratory of Pathology, West China Hospital. The ACD blood was centrifuged at 100 ×*g* for 10min to obtain platelet-rich plasma (PRP), and then diluted with physiological saline (ACD blood: physiological saline = l: 2.5). The HR of each heparinized scaffold was measured according to a procedure described previously ^3^ with some modifications. Briefly, the scaffolds were placed into separate test tubes. Physiological saline (10 mL) was added to the tubes and they were incubated at 37°C with shaking for 30 min. Diluted ACD whole blood (0.2 mL) was dropped into the vial at 37°C for 60min. Physiological saline and distilled water were used as negative and positive controls, respectively. The vials were centrifuged at 100 × *g* for 5 min and then the absorbance of the upper, clean solution was measured at 542 nm using a spectrophotometer (MQX200, BioTek, USA), as per the following formula:

HR (%) = (A1−A3) ∕ (A2−A3) × 100%

Where A1, A2, and A3 are the absorbance of the sample, the positive control, and the negative control, respectively.

**Reference**

1 Li, J., Zhu, W., Liu, J., Liu, X. & Liu, H. The hemocompatibility and the reabsorption function of TiO2 nanotubes biomembranes. *Chin Sci Bull* **57**, 2022-2028 (2012).

2 Xu, F. J., Li, Y. L., Kang, E. T. & Neoh, K. G. Heparin-coupled poly(poly(ethylene glycol) monomethacrylate)-Si(111) hybrids and their blood compatible surfaces. *Biomacromolecules* **6**, 1759-1768 (2005).

3 Liu, M. *et al.* Stabilized hemocompatible coating of nitinol devices based on photo-cross-linked alginate/heparin multilayer. *Langmuir* **23**, 9378-9385 (2007).
